# Ultrasensitive probing of plasmonic hot electron occupancies

**DOI:** 10.1038/s41467-022-34554-5

**Published:** 2022-11-05

**Authors:** Judit Budai, Zsuzsanna Pápa, Péter Petrik, Péter Dombi

**Affiliations:** 1 ELI-ALPS, ELI-HU Non-Profit Ltd., Szeged, H-6728 Hungary; 2 Department of Optics and Quantum Electronics, University of Szeged, Szeged, H-6720 Hungary; 3 Wigner Research Centre for Physics, Budapest, H-1121 Hungary; 4 Institute of Technical Physics and Materials Science, Centre for Energy Research, Budapest, H-1121 Hungary

**Keywords:** Nanophotonics and plasmonics, Optical techniques, Electronic properties and materials

## Abstract

Non-thermal and thermal carrier populations in plasmonic systems raised significant interest in contemporary fundamental and applied physics. Although the theoretical description predicts not only the energies but also the location of the generated carriers, the experimental justification of these theories is still lacking. Here, we demonstrate experimentally that upon the optical excitation of surface plasmon polaritons, a non-thermal electron population appears in the topmost domain of the plasmonic film directly coupled to the local fields. The applied all-optical method is based on spectroscopic ellipsometric determination of the dielectric function, allowing us to obtain in-depth information on surface plasmon induced changes of the directly related electron occupancies. The ultrahigh sensitivity of our method allows us to capture the signatures of changes induced by electron-electron scattering processes with ultrafast decay times. These experiments shed light on the build-up of plasmonic hot electron population in nanoscale media.

## Abstract (web summary)

Experimental evidence is given that upon the optical excitation of surface plasmon polaritons, a nonthermal electron population appears in the topmost domain of the plasmonic film directly coupled to the local fields.

## Introduction

Within the realm of plasmonics, it is of great interest to probe matter on the level of electron occupancies. The strong localization of plasmon assisted photon absorption facilitates the emergence of so-called hot electrons within a few tens of femtoseconds after plasmon excitation with energy levels that deviate significantly from Fermi-Dirac distribution. In recent years, much attention is focused on the ultrafast generation of these hot carriers, due to their fundamental role in emerging applications such as nanoscale electron emitters for ultrafast probing of matter^1^, construction of nanoscale optical circuitry^2–4^, hot electron-enhanced photocatalysis, photovoltaics and sensorics^5–7^. Regarding the feasibility of such devices, the development of theoretical and experimental methods to determine the energy distribution of hot electrons and to identify dominant mechanisms is essential. For localized plasmons in nanoparticles, Govorov and coworkers studied the generation and injection of plasmonic carriers from optically excited metal nanocrystals and they found that for nanocrystal sizes larger than 20 nm, the energies of excited electrons are close to the Fermi level^8^. The detailed theoretical study of Dubi et al. also pointed out that assuming continuous excitation, it is thermalization that dominantly determines electron distributions^9^. For a thin gold film that supports surface plasmon polaritons (SPPs), reflectometry experiments of Takagi et al. revealed slight differences in plasmonic response and temperature modulation spectra^10^. These differences may originate from electrons exhibiting a broader energy distribution belonging to the SPP mediated case, as shown in a comparative study of Heilpern et al. on photon absorption processes with and without SPP excitation via time-resolved reflectometry^11^. In a similar model system, Reddy et al. observed energetic electrons up to 0.3 eV based on transport measurements from single-molecule junctions^12^. However, all of these recent studies have focused on the energy distribution of the hot carriers, and only limited attention has been paid to the effect of the surface-bound nature of SPPs on the location of hot electron population in the depth of the film.

There are different SPP related phenomena exhibiting an in-depth variation. Upon SPP generation, a significant part of the electromagnetic energy of the incoming field is transformed to the kinetic energy of free carriers resulting in the longitudinal oscillation of electrons. The screening of the locally excess/absent negative charge carriers is described by the Thomas*-*Fermi screening, or more recently via the Friedel-oscillation both predicting that free carriers are confined within a few-nm vicinity of the surface^13^. This means that the excitation energy is dominantly concentrated near the surface^14^. During this process, known as SPP excitation, a collective oscillation of charges builds up with a confined, exponentially decaying electric field exhibiting also a field enhancement effect^15–17^. The volume of the SPP mode can be characterized by a penetration depth of ~10 nm into the metal. Within this volume, SPPs contribute to hot electron generation by various absorption mechanisms^18^. Phonon and defect assisted absorption occurs throughout the entire volume of the SPP mode in the metal, while carrier generation via Landau damping takes place right at the surface^19^. These local phenomena associated to SPPs affect the spatial distribution of hot electrons which is a fundamental question regarding hot-electron enabled devices. Although these theoretical results predict the existence of non-thermal electrons localized close to the sample surface, its experimental demonstration is still lacking. Thus, it is essential to develop an experimental method that is sensitive enough to explore the in-depth distribution of the electrons and identify the possibly appearing subdomains experimentally (referred to as layers of the plasmonic thin film throughout this paper) exhibiting different electron distribution functions.

Here, we demonstrate experimentally the existence of an electron population with high energies compared to the bulk of the gold film having a non-thermal nature; and more importantly, we also reveal its spatial extent near the surface of a plasmonic gold film. For the ultrasensitive probing of plasmonic electron occupancies we developed a spectroscopic ellipsometry method, enabling the detection of the energy distribution of the generated electrons.

In ellipsometry, measured data carry information about the changes in the polarization state of light upon reflection, which are quantified by two ellipsometric angles, conventionally called as *Ψ* and *Δ*, describing the relative amplitude and phase change of the reflected light. As these two quantities are in close correlation with the complex dielectric function, ellipsometry provides simultaneous access to the absolute value of both the real (*ε*_*1*_) and imaginary parts (*ε*_*2*_) of the dielectric function. As electron occupancies change during the photoexcitation of metals, so does the dielectric function^20^. This means that it is possible to retrieve electron occupancies related to plasmon excitation and decay by measuring the dielectric function of the plasmonic system in a highly sensitive way. Another advantage of this approach is its ultrahigh sensitivity to surface phenomena^21^ and the in-depth information about the dielectric function of the layers constituting the gold film. By exploiting these features of ellipsometry, we could reveal that continuous wave (cw) excitation holds fingerprints of several plasmon-related effects simultaneously.

## Results

### Experiments

**Fig. 1:**
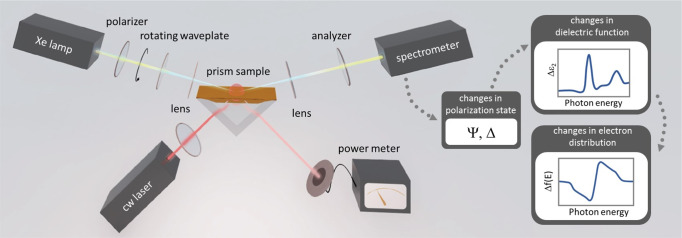
Experimental setup for ultrasensitive probing of plasmonic hot electrons with spectroscopic ellipsometry. SPPs are excited by a cw laser at the resonant angle of incidence from the backside, while ellipsometric data is recorded on the gold-air interface with white-light probing from the top. These measurements are capable of accessing the dielectric function of the investigated system, the changes of which are in close correlation with the electron distribution function.

For the excitation of SPPs, we applied the Kretschmann geometry involving a glass right-angle prism coated with 45 nm gold by thermal evaporation (see Fig. 1 and Methods section).

**Fig. 2:**
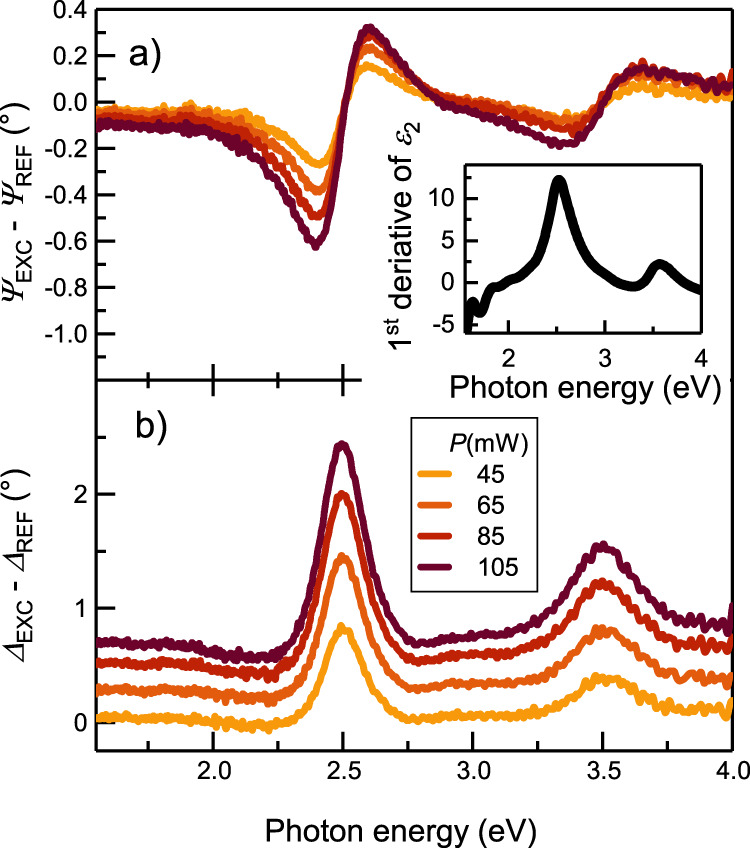
Measured ellipsometric difference curves for surface plasmon polariton excitations at different laser powers. **a** Differences in psi spectra and (**b**) differences in delta spectra. Note that delta values (**b**) are offset for better visibility. The inset shows the first derivative of the imaginary part of the dielectric function of gold determined in this study. Peak positions in the ellipsometric curves correspond to rapid changes in the dielectric function of gold, i.e. peaks in its first derivative.

We applied different laser intensities for exciting SPPs, and, as a unique feature, monitored the changes in the optical properties of the plasmonic system by recording the ellipsometric response with and without plasmon excitation (i.e. at the on and off states of the cw laser that excites plasmons, respectively), the latter for reference. Although the ellipsometric angles of the non-plasmonic geometry and the one inducing SPPs differ only very slightly (see Supplementary Information), notable deviations can be identified in the difference signal (presented in Fig. 2a, b) with blue curves noted as (*Ψ*_*EXC*_*-Ψ*_*REF*_ and *Δ*_*EXC*_*-Δ*_*REF*_). Though the amplitude of the emerging peaks increases with increasing laser power, their position can not be directly correlated with the excitation energy (1.53 eV), as one would expect from classical spectroscopic methods. Instead, as a matter of fact, it is related to the structure of the dielectric function near the spectral regions where it changes rapidly (photon energies of band-to-band transitions occurring as structures in the first derivative of *ε*_*2*_, see inset of Fig. 2). This clearly indicates that the observed differences in the ellipsometric curves originate from the changes in the dielectric function, with the change clearly being induced by SPP generation. To follow these trends quantitatively, it is necessary to perform detailed modeling.

### Retrieving the dielectric function

**Fig. 3:**
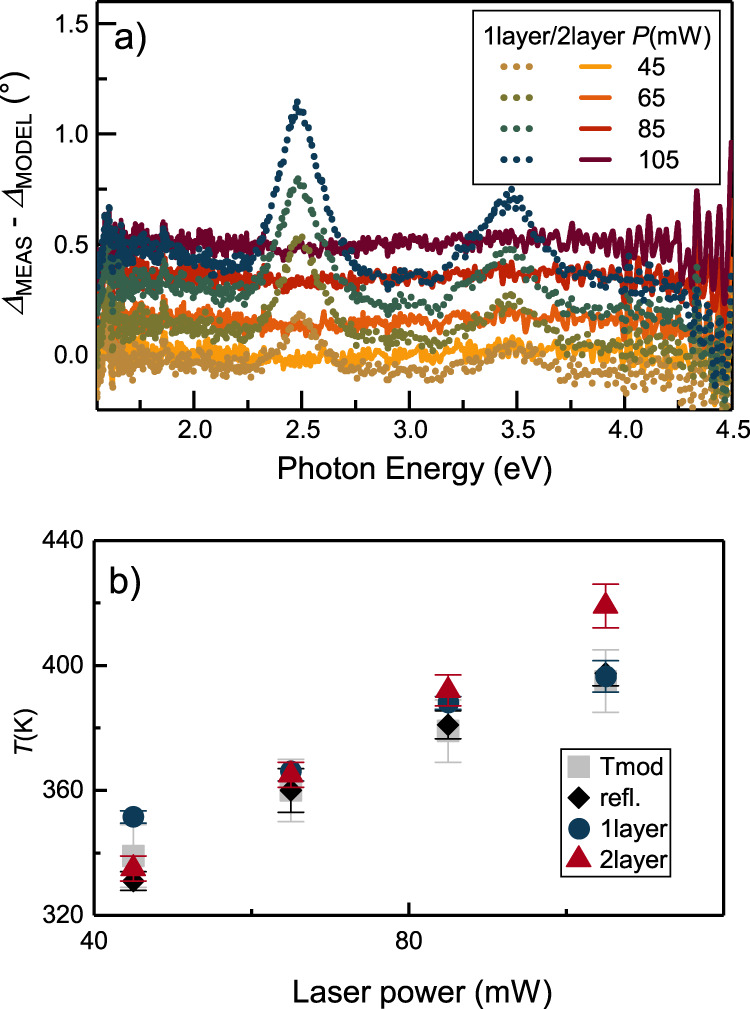
Applicability of the different ellipsometric models. **a** Comparison of the measured and modeled ellipsometric data. Dotted curves belong to the single layer model, where the appearing distinct peaks provide a clear indication that this simple model is not able to describe the measured changes sufficiently. Solid curves with no modulation belong to the two-layer model, which reproduces perfectly the measurements. **b** Temperature of the thin film hosting the SPPs as a function of laser power applied for SPP excitation. Gray squares, black diamonds, dark blue circles and red triangles represent the temperature originating from temperature calculations, from reflectivity measurements, from the ellipsometric model considering a single layer with elevated temperature, and from the ellipsometric model considering two layers, respectively. Error bars of the temperature calculations are defined by the uncertainties of the power converted to SPP excitation; for reflectivity measurements, the uncertainty of *C* parameter was taken into account; while for the ellipsometric results, error bars originate from thickness uncertainties (standard deviations of the deduced results in all cases).

Ellipsometric modeling includes the description of the layer structure of the investigated system, and more importantly the optical behavior of each layer. In our cw excitation case, one would expect that the most prominent effect is the temperature rise of the whole film, due to the mechanisms accompanying the decay/absorbance of SPPs. If it is only the temperature that initiates measurable changes in the ellipsometric response of the sample upon SPP excitation, then a model system containing a single homogeneous gold layer with temperature-dependent dielectric function should describe our data perfectly^22^. For determining the temperature-dependent dielectric function of gold, we performed ellipsometric measurements on a uniformly heated sample (containing the same gold layer as the gold coated prism), without SPP excitation (details about ellipsometric modeling is provided in Supplementary Information). The resulting temperature dependent dielectric function datasets were used to describe ellipsometric data gained upon SPP excitation. When calculating the deviation of the modeled ellipsometric data and the measured ones, distinct peaks occur – see e.g. the difference in the delta curves in (Fig. 3a) -, clearly indicating that solely the temperature rise is not able to sufficiently describe the measured changes. In other words, there must be other contributions beside the temperature increase of the plasmonic thin film originating from the absorption of SPPs near the surface.

To prove that this additional non-thermal contribution belongs exclusively to SPP excitation, we performed measurements by illuminating the sample from the top, so that SPP generation is absent. It can be shown that the ellipsometric difference curves belonging to top illumination behave similarly to the ones recorded on uniformly heated sample, and are clearly distinguishable from the plasmon mediated case (Supplementary Information). This supports that SPP related effects have to be taken into account to describe the observed changes.

The direct effects of the presence of SPPs can be twofold. (i) Nonlinear effects can take place if the enhanced near-fields exceed a certain value^23^, and (ii) the occupancy of the electronic states is altered upon plasmon excitation^9,11^. By determining the local field distribution, we can exclude the nonlinear effects caused by the presence of enhanced plasmonic near-fields, as the emerging near-fields are not high enough (Supplementary Information). Focusing onto the modified occupancy of the electronic states, we have to take into account that SPP-related phenomena appear at the surface. Therefore, the non-thermal electron distributions associated to SPPs affecting the dielectric function might be present only in the topmost portion of the gold film, similarly to the penetration effect already noted for direct photon absorption by Heilpern et al.^11^. Furthermore, due to the energy dependent characteristic times of the decay processes, we can expect that electrons with different energy levels exhibit different spatial patterns within the gold film, and the more energetic, non-thermal electrons are located close to the sample surface^6^. As a consequence, we divided the gold film into two layers in our model, (i) a lower layer with increased temperature exhibiting thermal electron distribution (thermalized layer), described via the temperature dependent optical model as before, and (ii) an upper layer accounting for the non-thermal electron distribution due to the appearance of SPPs and the associated hot electron generation (non-thermalized layer). When calculating the deviation of the measured and simulated ellipsometric curves, the distinct peaks occurring earlier in the case of the simple thermal model diminish (Fig. 3a). This plot clearly shows that the model properly describes the measured data, and supports the presence of the non-thermalized surface layer in accordance with the theoretical predictions.

Both modeling procedures allowed us to estimate the temperature of the gold film, values of which are shown in Fig. 3b with dark blue circles for the purely thermal model (single layer model) and red triangles for the non-thermal modeling (two-layer model). For the latter case, the temperature for the thermalized part of the gold film is shown. We compared these temperature values with the results of two other temperature estimations, (i) an experimental method based on the reflectivity changes of the gold film and (ii) temperature calculations. Determining the local temperature of metal thin films is not straightforward, therefore we applied the method of measuring the temperature dependent changes in the reflectivity of metals in the visible spectral range^24^, which can be applied by means of the ellipsometric measurements as well (see Supplementary Information). This way, we could determine the temperature of the gold film under SPP excitation (Fig. 3b), black diamonds. Thermal modeling carried out with COMSOL Multiphysics (see Supplementary Information) supports these values, as evidenced by (Fig. 3b), gray squares. As a further validation of our modeling, temperature values of all four approaches coincide within the error bars.

In the final section of the paper, we are focusing on the conclusions drawn from the two-layer model and their interpretation. As the main results of the ellipsometric analysis, the thickness and the dielectric function of the non-thermalized and thermalized layers became also available. The thickness of the non-thermalized layer increases with the applied laser intensity from 4 nm to 6 nm corresponding to the spatial extent of the hot electron population. Here we have to note, that though our modeling would suggest a step-like transition between the spatial regions of non-thermal and thermal carriers, in reality the boundary between these regions follows a continuous transition. Such a continuous change in the electron distributions could have been mimicked as a gradient with several sublayers but such description would result in a large number of fitting parameters and thus large uncertainties. To avoid these, we applied the simplest, step-like model, which – based on the results – already provides a clear indication that non-thermal carriers are located close to the surface of the gold layer in a nanometric volume.

**Fig. 4:**
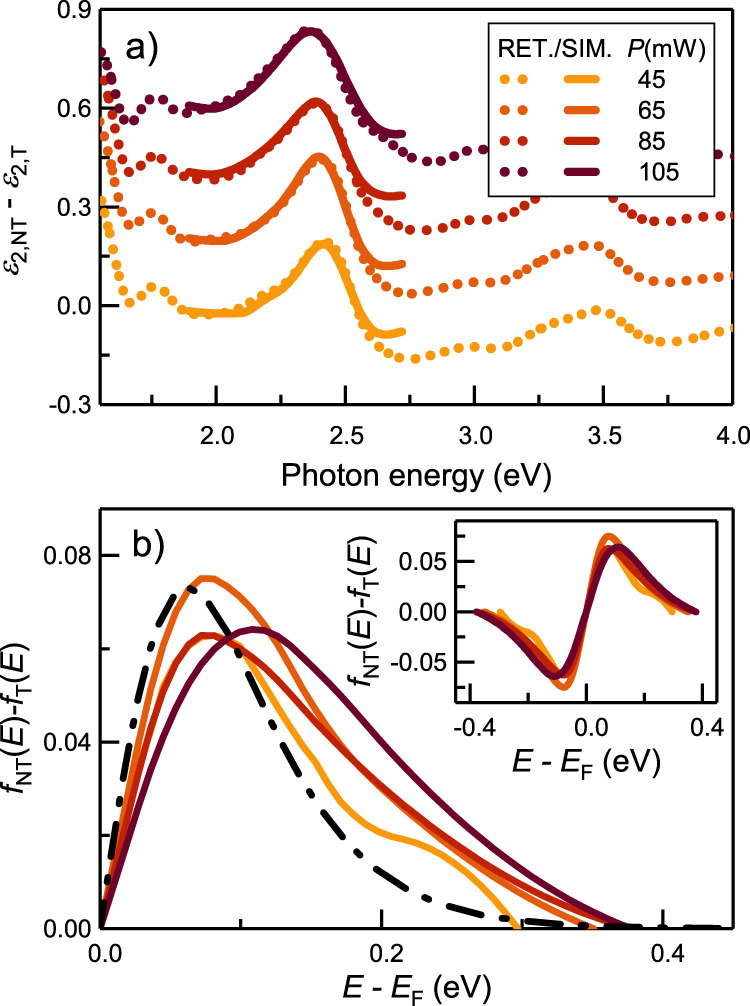
Differences in the dielectric function of the non-thermalized and thermalized layers and the corresponding differences in the electron occupancies. **a** Retrieved (set of dotted curves) and simulated (set of solid curves) differences between the dielectric function of the non-thermalized top layer (NT) and the thermalized part (T) of the gold film and (**b**) the corresponding changes in the electron occupancies above Fermi-level. The inset shows Δ*f(E)* in the whole affected energy range. For comparison, the difference of two Fermi-Dirac distributions belonging to Δ*T*= 275 K is presented with a dash-dotted line.

When comparing the dielectric function of the thermalized and non-thermalized layers, minor differences can be observed in the intraband transition part of the spectrum (below 1.7 eV) with major differences occurring in the interband transition domain. In the intraband domain, the imaginary part of the dielectric function at the surface is slightly increased as compared to that of the thermalized part (Fig. 4a), while in the interband region, there are multiple features with the most prominent peak appearing in the difference curves around 2.3 eV. These features become wider as the applied laser intensity increases. To gain insight into the origin of these differences, first we applied a different excitation geometry, which allowed us to exclude the contribution of nonlocal effects^25–27^ (for details see Supplementary Information). As a next step, we calculated the dielectric function of the two layers based on its proportionality with the joint density of electron states and electron occupancies. Practically, we considered different transitions near the X and the L points of the Brillouin-zone and different electron occupancies (*f(E)*) to reproduce the most prominent peak of the ε_2_ difference curves (Δ*ε*_*2*_). Description of other features of the Δ*ε*_*2*_ curves corresponding to further transitions are beyond the scope of this work. To avoid any presumption regarding the Δ*f(E)* curves, we introduced a spline-based fitting method capable of the direct inversion of the Δ*ε*_*2*_ curves (for details see Supplementary Information). Fitting of the 2.3 eV peak of the Δ*ε*_*2*_ curves and the resulting Δ*f(E)* curves are presented in Fig. 4. along with the Δ*f(E)* curve belonging to two Fermi-Dirac distributions having temperatures of 400 K and 550 K as a reference, for the corresponding purely thermal Δ*ε*_*2*_ curve see Supplementary Information.

According to the curves in (Fig. 4b), we could identify changes in the electron distribution only in the vicinity of the Fermi-level in all cases, suggesting that the number of energetic hot charge carriers is very low in accordance with Ref. 8. With increasing laser power, the Δ*f(E)* curves differ from the thermal distribution to an increasing extent: the curves become broadened with a slight shift in their maxima. Both broadening and spectral shift can be the consequence of an increased electron temperature of the top layer taking into account an elevated background temperature. An increase in the electron temperature increases the plasma frequency and reduces the electron scattering lifetimes^28^, resulting in slightly higher infrared absorption and also influencing the occupancies in the vicinity of the Fermi-level (Fig. 4b)^11^. However, the extent of the broadening (mainly for the higher laser powers) indicates a more distorted electron distribution. This is the spectral fingerprint of an additional hot electron population with energy levels up to 0.4 eV measured from the Fermi level. Upon the generation of SPPs with photons of *ħω* energy, their phonon and surface assisted absorption results in charge carriers with average excess energies of *ħω*/2, electron-electron scattering assisted absorption generates electrons of lower average energy, namely *ħω*/4^14^. This energy is equal to 0.4 eV in our case, indicating that electron-electron scattering assisted absorption is the dominant mechanism. Interband absorption would excite electrons just above the Fermi energy, but this process is not considered here due to the low photon energy applied (*ħω* = 1.53 eV).

The retrieved energy distribution of electrons in the thermalized and non-thermalized layers can be interpreted as follows. At the surface, SPPs are continuously generated, and they are absorbed within a few tens of femtoseconds via different mechanisms, resulting in electrons (and holes) of elevated energies. These energetic electrons thermalize via electron-electron and electron-lattice scattering, approaching an equilibrium, i.e. reducing the electron energies and increasing the lattice temperature of the whole film as we see in the thermalized part of the gold film. More interestingly, our observations provide a clear indication of an additional hot-electron population close to the film surface within a nanometric range depending on the excitation intensity. The extent of this non-thermalized layer coincides with the expected location of hot electron generation via Landau damping which would result in electron energies up to 0.8 eV (*ħω*/2) with respect to the Fermi level. However, since the electron-electron scattering probability is strongly dependent on energy, - namely electron collision rate is much larger for more energetic electrons^9^, - less energetic electrons remain in the system giving rise to the observed electron distribution in the 0.4 eV vicinity of the Fermi level.

In summary, we could detect the development of a hot electron population in a nanometric surface layer induced by the continuous excitation and absorption of SPPs. The signatures of these transient electronic states of hot electrons were revealed with the help of spectroscopic ellipsometry. Taking advantage of the in-depth information extracted with our method, we could decouple the heating effect of plasmon decay from the direct effect of SPPs on the occupancies of electronic states. This enabled us to directly probe and demonstrate the presence of plasmonic hot charge carriers at the surface. The method demonstrated here also opens the pathway toward ultrafast time-resolved investigations of hot electron dynamics with ultrasensitive optical tools, i. e. with ultrafast spectroscopic ellipsometry.

## Methods

### Experimental methods

For the excitation of SPPs, we applied the Kretschmann geometry involving a right-angle glass prism coated with 45 nm gold layer prepared in a custom-made Pfeiffer Vacuum Classic 570 coating chamber by thermal evaporation, under the following conditions: The chamber was pumped by a HiPace 1500 turbopump with 1450 l/sec. The base pressure was 2×10^−5^ mbar (1.5×10^−5 ^Torr). The source to substrate distance was 35 cm. A planetary substrate holder was used to reach homogeneous film thickness. A 70 mm long by 17 mm wide by 0.5 mm thick (Umicore) tungsten boat was used with 1 g 99.99% Au pellets as source material. The boat was heated by a 4 V, 1000 A power supply. The deposition rate was 10 Å/s. With these parameters the rms roughness of our gold layer is 0.6 nm (measured with atomic force microscope), which was taken into account in the analysis. Compared to Norris recipe^29^, the parameters are very similar except for the base pressure, which was limited by the chamber properties. The differences in base pressure explain our slightly larger rms roughness value as compared to that measured by Norris and coworkers. They reported 0.3 nm rms roughness 10 Å/s deposition rate and 3×10^−8^ Torr base pressure, and 0.4 nm rms roughness for 0.5 Å/s and 2 × 10^−6^ Torr.

The list of the different phases/layers of our measured system is shown in Table 1.

**Table 1:** Different phases/layers of the investigated plasmonic system and their thicknesses

Phase/layer	Thickness
air	∞
roughness	0.6 nm
gold – non-thermal	4-6 nm
gold – thermal	39-41 nm
chromium	2 nm
glass substrate	∞

SPPs were excited from the backside of the film using an 808 nm (1.53 eV) cw diode laser illuminating the sample under the angle of a sharp SPP resonance. Simultaneously, spectral fingerprints of SPP-related changes were monitored by means of spectroscopic ellipsometry (Semilab SE1000, rotating compensator spectroscopic ellipsometer), illuminating the sample from the top side (i.e. directly the air-gold interface) with a continuous, broadband light source (Xe lamp, applied photon energy range: 1.55-4 eV) under 65° angles of incidence. The light from the Xe lamp is in a well-defined polarization state being set by a fixed polarizer and the rotating waveplate/compensator. The polarization state after reflection from the sample is monitored by another fixed polarizer, called analyzer.

### Computational methods

Different ellipsometric models were used to describe the actual properties of the sample under the various excitation conditions. These are detailed in the Supplementary Information, here we provide only a short overview. To determine the thickness of our gold film we applied a five-component model consisting of BK7 glass, Cr adhesion layer, gold layer, surface roughness (determined by atomic force microscopy) and air. We described the optical properties of the gold layer and the surface roughness layer with a spline-based optical model, and with effective medium approximation, respectively. Besides its dielectric function, spline-based model provided also the thickness of the gold film. Modeling of the gold film hosting SPPs were carried out with i) a single layer model, handling the gold as a homogeneous film using the retrieved temperature dependent optical properties, while (ii) the 2-layer modeling applying the temperature dependent optical data to describe the thermalized layer and the optical properties of the non-thermalized layer were described using again the spline based optical model. The total thickness of the layer system was kept fixed, and several iteration steps were done to find the optimal thickness of the non-thermal layer showing the best fit.

To reveal the origin of the differences found when comparing the non-thermalized and thermalized layers’ dielectric constants, we calculated the dielectric function based on its proportionality with the joint density of electron states and electron occupancies^11^:

1 $${\varepsilon }_{2}\left(\omega \right)={\varepsilon }_{2,{intra}}\left(\omega \right)+\frac{A}{{({{\hslash }}\omega )}^{2}}\int _{{E}_{{\min }}}^{{E}_{{\max }}}D\left({{\hslash }}\omega,\,E\right)\left(1-f\left(E\right)\right){dE},$$

where *ε*_*2,intra*_ is the contribution of the intraband electronic transitions, $D\left(\hslash \omega,{E}\right)$ denotes the energy distribution of the joint density of states (EDJDOS), *f(E)* describes the electron energy distribution. Constant *A* was determined based on a fitting to the dielectric function measured in this study at room temperature without laser excitation. To determine *ε*_*2,intra*_ a Drude function was applied^28^, while to calculate the EDJDOS parabolic band structures were assumed at the L and X points in the Brillouin zone according to^11,30^. The changes in the electron distribution function were modeled with a spline-based fitting algorithm avoiding any a priori assumptions.

## Supplementary information

Supplementary Information

## Data Availability

The data that support the findings of this study are available from the corresponding author on request.
