# Supplementary material for: Ultrasensitive probing of plasmonic hot electron occupancies
Source: Nat Commun. 2022 Nov 5;13:6695. doi: 10.1038/s41467-022-34554-5 (PMC9637162; doi:10.1038/s41467-022-34554-5)
Supplement: Supplementary file 1 — Supplementary Information [file 41467_2022_34554_MOESM1_ESM.pdf]

Supplementary Information for „**Ultrasensitive probing of plasmonic hot electron occupancies**"

J. Budai[1,2], Z. Pápa[1,3], P. Petrik[4], P. Dombi[1,3]

1 ELI-ALPS, ELI-HU Non-Profit Ltd., Szeged, H-6728, Hungary

2 Department of Optics and Quantum Electronics, University of Szeged, Szeged, H-6720, Hungary

3 Wigner Research Centre for Physics, Budapest, H-1121, Hungary

4 Institute of Technical Physics and Materials Science, Centre for Energy Research, Budapest, H-1121, Hungary

* corresponding author: Judit Budai, judit.budai@eli-alps.hu

1. Spectra of the measured ellipsometric angles

Ellipsometric angles are defined as the amplitude ratio (Ψ) and the phase difference (Δ) between the two linearly polarized components (p and s) of polarized light illuminating the sample at non-normal incidence (p component is parallel with the plane of incidence and s component is perpendicular to it). When applying different laser powers for exciting surface plasmon polaritons (SPPs), typical Ψ and Δ spectra could be recorded for gold films (Fig. S1), which varied with the applied laser power only slightly.

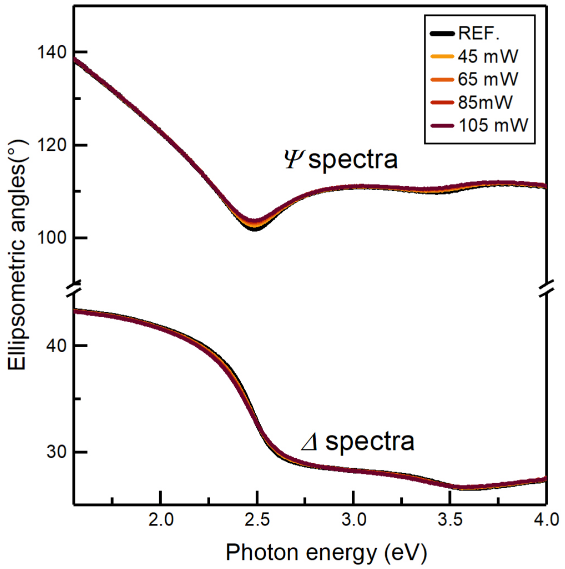

Fig. S1. Ellipsometric angles as a function of photon energy measured upon excitation with different laser powers.

2. Details of the ellipsometric modeling of the plasmonic gold film

As a first step, the room-temperature (RT) data without laser excitation was analyzed.

We described the dielectric function of RT gold film with a spline based optical model [1]. The B-Spline model describes ε_2 by first defining evenly spaced photon energies, which are used as control points (knots or nodes) and by interpolating the optical constants between these control points. The ε_1 curve is calculated from the ε_2 curves with the help of Kramers-Kronig integration. In our case, the spacing between the nodes was set to be 0.15 eV. To allow for Kramers-Kronig integration, two further nodes were defined outside the measured photon energy range at a 'distance' of 0.5 eV from the lower and the upper limit. To handle the effect absorption outside the investigated measurement range the amplitude of a zero oscillator located at low photon energies was fitted along with the integration constant.

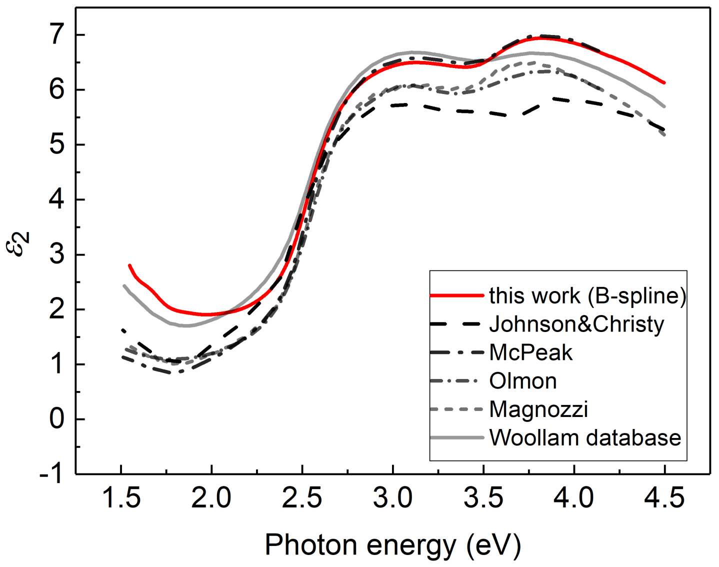

Fig. S2. Comparison of ε_2 of gold determined in this work with literature data [2-6].

Using this dispersion law, and using a five-component model, namely BK7 glass, Cr adhesion layer, gold layer, surface roughness (determined by atomic force microscopy) and air, the thickness of Cr adhesion layer and gold layer could be determined. The modeling also provided the dielectric function of our gold film at room temperature. The resulting ε_2 curve is plotted in Fig. S2 in comparison with literature data [2-6].

During the evaluation of the spectra recorded at higher temperature (100°C), the thickness values were kept fixed and only the dielectric function was fitted to deduce the optical data. This way we could set up a tabulated dielectric function dataset characterizing the temperature dependence of our gold layer. Later to determine the dielectric function of our gold film at any arbitrary temperature linear inter/extrapolation of this dataset was used assuming linear changes in the dielectric function in this temperature regime as supported by [7]. This model allowed us to set the temperature of the gold layer as fitting parameter.

In the model describing the case when SPPs are present, we divided the gold film into two sublayers, a lower layer having the temperature dependent optical model as described above (thermalized layer), and an upper layer accounting for the appearance of SPPs (non-thermalized layer). The optical properties of the non-thermalized layer were described using again the spline based optical model. The total thickness of the layer system was kept fixed. To determine the thickness of the non-thermalized layer, several different thickness values were set during the fitting procedure, during which the optical properties of the non-thermalized layers were fitted along with the temperature of the underlying thermalized layer, and the fit quality (mean squared error of the measured and fitted curves - *MSE*) was determined [8]. For all excitation powers, an optimal thickness could be identified, where the preset thickness resulted in a minimal *MSE* (Fig. S3). In addition to the physical considerations, the existence of such minima validates the application of the two-layer model.

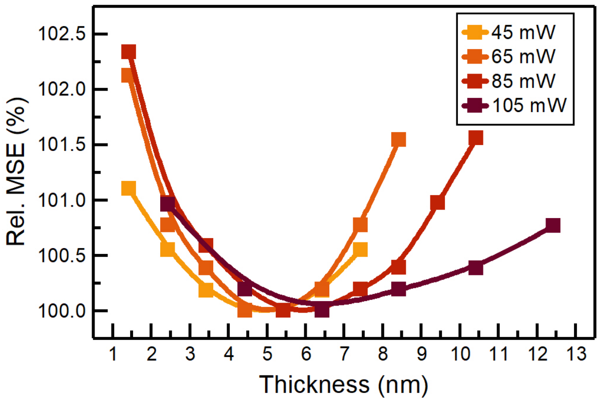

Fig. S3. Changes in the fitting quality when setting different thicknesses of the upper layer in the two-layer model. For all excitation powers, an optimal thickness could be identified, where the preset thickness resulted in a minimal MSE.

3. Comparison with topside illumination

To further prove that the non-thermalized layer exists only in the case of plasmon excitation, we performed a third type of measurement, where the gold film was illuminated from topside at normal incidence (SPP excitation is excluded). The difference curves in this case are compared to those measured upon uniform heating and upon SPP excitation of the sample (Fig. S4 - for comparison the measured difference curves are normalized.) The first two cases do not promote SPP excitation, their normalized difference curves behave similarly, and the observed changes can be attributed to the temperature rise within the sample.

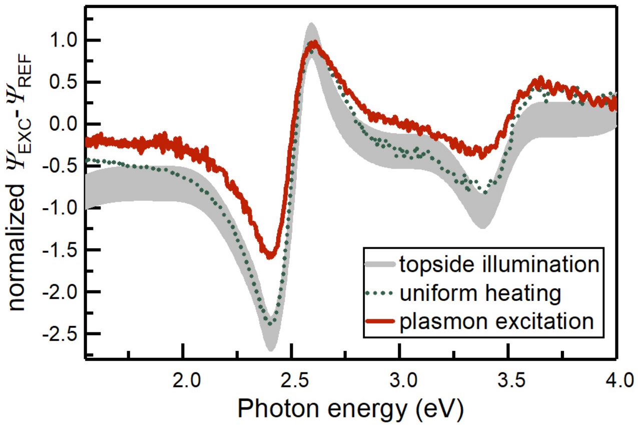

Fig. S4. Normalized ellipsometric difference spectra measured in the case of topside laser illumination (grey area showing the average of several laser power excitations), uniform heating of the sample (dotted green line) and plasmon excitation (dark red blue line). Curves belonging to topside excitation and uniformly heated sample show similar difference spectra clearly distinguishable from the plasmon excitation case.

4. Temperature of the gold film upon SPP excitation

The temperature rise of the sample upon SPP excitation was estimated additionally using two independent methods: a measurement based on reflectivity change of the gold and a simulation tool. We discuss these methods in the following sections.

4.1 Performing thermoreflectivity-based temperature estimation with ellipsometry

A broadly applied method for measuring the temperature of metal thin films is based on the measurement of the temperature dependent changes in the VIS reflectivity of metals according to the following formula [9]:

$$\frac{\Delta R}{R} = C \cdot \Delta T, \qquad \text{(S1)}$$

where *ΔR* is the reflectivity change of the sample between two states exhibiting a temperature difference of *ΔT*, and *C* is a constant.

To determine the relative reflectivity increment from the recorded ellipsometric data we can directly use the recorded average intensities. First, the constant *C* belonging to our samples was determined from ellipsometric measurements, during which the samples were heated to preset temperatures. Then the deduced value of *C* was applied to evaluate the temperature of the samples during SPP excitation by using the S1 expression. To validate our ellipsometric approach, we compared the deduced *C* parameter with literature values [9-12] and found very good agreement (Fig. S5).

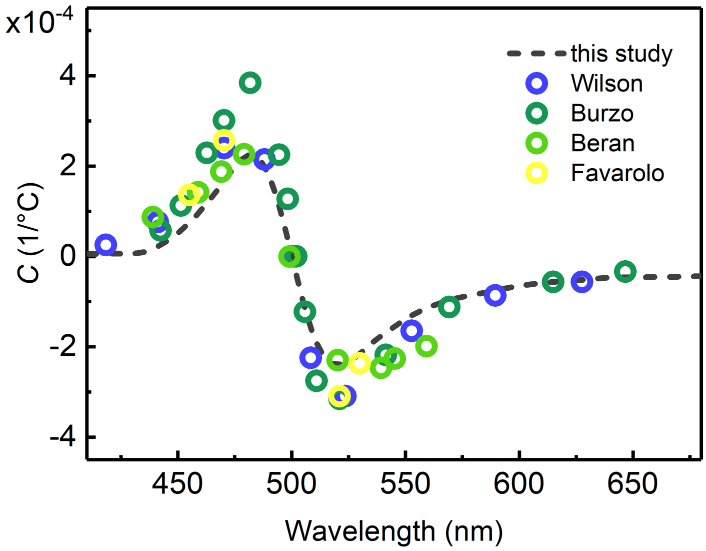

Fig. S5. Comparison of *C* parameter deduced from our measurements with literature data [9-12].

4.2. Temperature calculations

To support that the effect of SPP excitation is not a thermal gradient within the layer but the measured data can only be interpreted as an additional electron population with a non-Fermi-Dirac energy distribution, we carried out thermal modeling using COMSOL Multiphysics. For the calculations, we considered that the laser power that is spent on plasmon excitation is absorbed in the top boundary of the gold layer (gold-air interface). For this in our SPP excitation setup, we measured the power of the incoming laser beam and the power of the reflected beam both in s- and p-polarization states. The difference between these two polarizations will account for surface plasmon excitation, since SPPs are generated only in p-polarization state, while with the comparison with s-polarization we can take into account the possible losses due to e.g. reflections on the different interfaces. In short, with this estimation, we assume that all the power - that is not reflected - will excite plasmons, and all the power of this plasmon excitation will then be converted to the heating of the gold.

In our simulation, this absorbed power is fed to a heat source located at the top 5 nm of the gold layer. With such location of the heat source, we tried to mimic the field localization property of the SPP generation, namely that a significant part of the electromagnetic energy of the incoming field is transformed to the kinetic energy of free carriers, which are confined within a few-nm vicinity of the surface (due to Thomas-Fermi screening or Friedel-oscillations).

The modeled part of our investigated system contains a 10 mm thick glass substrate, 45 nm gold layer on the top and the surrounding air. The simulation domain is 3D with cylindrical symmetry. The modeled cylindrical volume has 4 mm diameter with 260-μm diameter heat source representing the laser spot.

The resulting steady-state temperature distribution shows negligible difference between the top and the bottom part of the gold layer (ΔT<0.1 K) supporting that the measured signal is not the consequence of a temperature gradient. Furthermore, the calculated temperatures of the gold layer for the different applied laser power values coincide with the ones deduced from the reflection based and from the ellipsometric measurements within the error bars (see Fig. 3 of the main article).

5. Effect of SPP electric field on the optical properties

To reveal the possibility of nonlinear effects on the optical properties, we considered the maximal intensity on the surface of the gold film taking into account also the plasmonic field enhancement effect, and calculated the nonlinear refractive index using the available nonlinear optical parameters from the literature.

In our excitation geometry, the intensity is 131.25 W/cm^2 for the largest applied laser power. We calculated the electric field distribution in the direction perpendicular to the sample surface using Lumerical FDTD Solutions software under the same conditions applied during our experiments. Field enhancement factor as high as 15 could be obtained near the surface, resulting in $3*10^4$ W/cm^2 =$3*10^8$ W/m^2 enhanced intensity (Fig. S6).

The nonlinear refractive index can be estimated with the following formula: $n=n_1+n_2*I$, where n_1 is the linear refractive index, and n_2 is the term characterizing the nonlinearity in m^2/W units. I is the intensity. Since the literature values of the n_2 for bulk gold range from 10^{-12} to 10^{-16} m^2/W [13], the nonlinear term has a negligible - at least 4 orders of magnitude smaller than the linear term - contribution in our case. Moreover, since the field strength inside the gold film drops significantly and decays within a few tens of nanometers, the effect is expected to be even smaller.

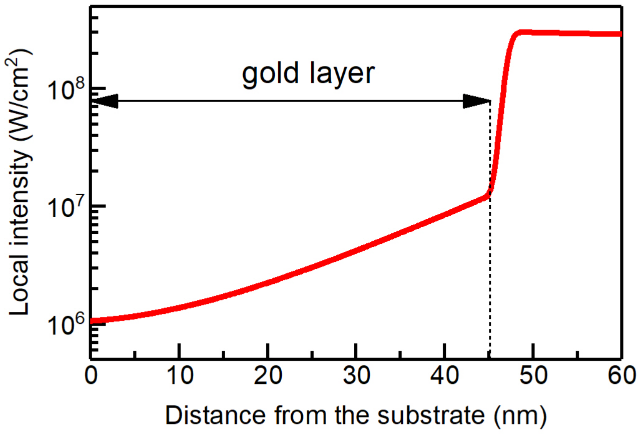

Fig. S6. Local intensity in our excitation geometry inside the gold film and in its vicinity considering also the field enhancement property of the generated SPPs.

6. Excluding possible nonlocal effects

One would expect that the observed changes can be attributed to nonlocal effects. However, according to [14, 15] nonlocality manifests in anisotropic behavior of the dielectric function. To test the possibility of nonlocal effects, we carried out our measurements when the plane of the ellipsometric setup is parallel or perpendicular with respect to the propagation direction of the SPPs. The measured ellipsometric difference curves show no deviations depending on the excitation geometry (Fig. S7). This supports that no anisotropic effects have to be taken into account.

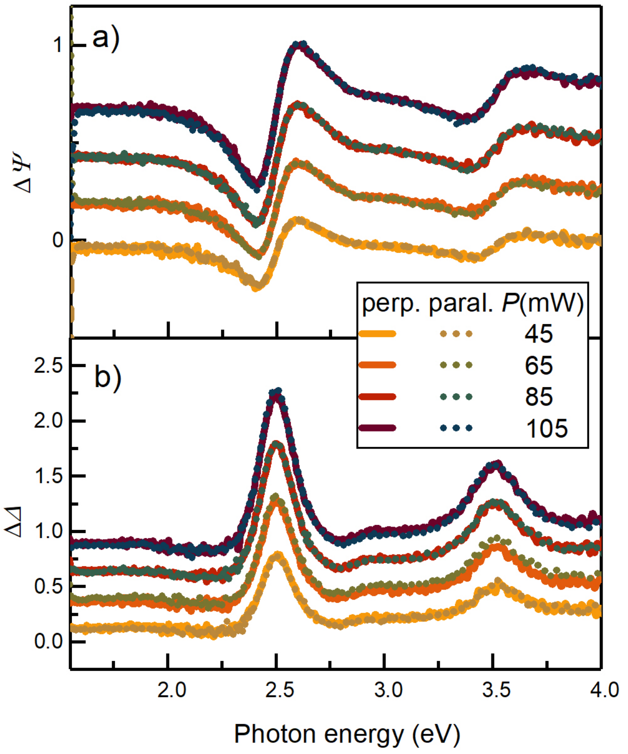

Fig. S7. Measured ellipsometric difference curves in different excitation geometries when the plane of the ellipsometric setup is parallel or perpendicular with respect to the propagation direction of the SPPs.

7. Changes in the electron distribution

7.1. Parameters for EDJDOS calculation

For calculating the EDJDOS, parabolic band structures were assumed at the *L* and *X* points in the Brillouin zone with the following band energies and masses (sources included). With these parameters the interband contributions and resulting ε_2 curve is shown in Fig. S8.

	X_u	$m_{u\parallel}$	$m_{u\perp}$	X_l	$m_{l\parallel}$	$m_{l\perp}$	*source*
X	1.52 eV	0.12 m_e	0.24 m_e	-1.58 eV	1.03 m_e	0.7 m_e	[16, 17]
L	-0.74 eV	0.25 m_e	0.22 m_e	-2.28 eV	0.81 m_e	0.87 m_e	[17, 18]

Table S1. band structure parameters used for calculating EDJDOS.

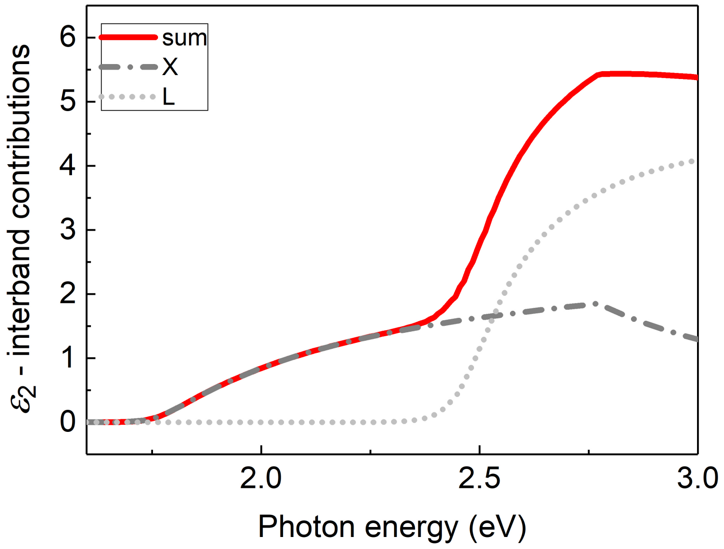

Fig. S8. Interband transitions considered during the analysis

7.2. Inversion of *f(E)*

In our representation, we did not focus on the absolute values of the electron distributions, but on its changes. Δ*f(E)* can be related to the changes in the dielectric function which can be obtained from Eq (1) through simple mathematical steps:

$\Delta\varepsilon_2(\omega) = \Delta\varepsilon_{2,\,intra}(\omega) + \frac{A}{(\hbar\omega)^2}\int_{E_{min}}^{E_{max}} D(\hbar\omega,\ E)\Delta f(E)\,dE$, where $\Delta\varepsilon_2$ denotes in our case the difference between the imaginary part of the dielectric functions of the top non-thermal and the bottom thermal layer, while the $\Delta\varepsilon_{2,\,intra}$ term describes the changes in the intraband transitions handled with a Drude function.

Upon the evaluation of the retrieved $\Delta\varepsilon_2$ data we deduced the Δ*f(E)* curve with a simple spline based fitting method. In this method, first we define energies where the actual value of the Δ*f(E)* curve can be adjusted (knots or nodes). Second, between these energy values the Δ*f(E)* curve is interpolated with the help of a quadratic spline. As a third step, we adjust the Δ*f(E)* values at the knots with a fitting algorithm, to describe the previously retrieved $\Delta\varepsilon_2$ curves in the 1.8-2.7 eV range.

7.3 Comparison with thermal effects

To analyse how the retrieved Δ*f(E)* curves relate to Δ*f(E)* curves belonging to pure thermal effects, we analyzed purely thermal $\Delta\varepsilon_2$ curves calculated from literature data [5] (providing access to datasets measured at higher temperatures) by using two Fermi-Dirac distributions. The direct comparison of thermal and non-thermal (plasmon-assisted) $\Delta\varepsilon 2$ curves shows already rather important differences: the shape of the 'baseline' is rather different indicating a different change in the free electron properties, i.e. Drude terms/intraband contribution. Furthermore, the shape of the main peak at around 2.3 eV is wider for the non-thermal case. For the purely thermal case, the base temperature was set to the lattice temperature belonging to the highest laser intensity of the plasmon excitation case (105 mW). The temperature difference was set so that the amplitude of the Δ*f(E)* curves retrieved at 105 mW coincide with that calculated from the two Fermi-Dirac curves. From the point of view of the reliability of our method, the purely temperature dependent reference $\Delta\varepsilon_2$ data could be well described with the help of the Fermi-Dirac distributions when taking into account a slight shift in the band structure for the higher lattice temperature due to lattice expansion during heating [16]. Regarding the shape of the Δ*f(E)* curves, the extent of the broadening is much larger for the highest laser intensity than that of a thermal system, holding the spectral fingerprint of an additional hot electron population with energy levels up to 0.4 eV measured from the Fermi level (Fig. S9).

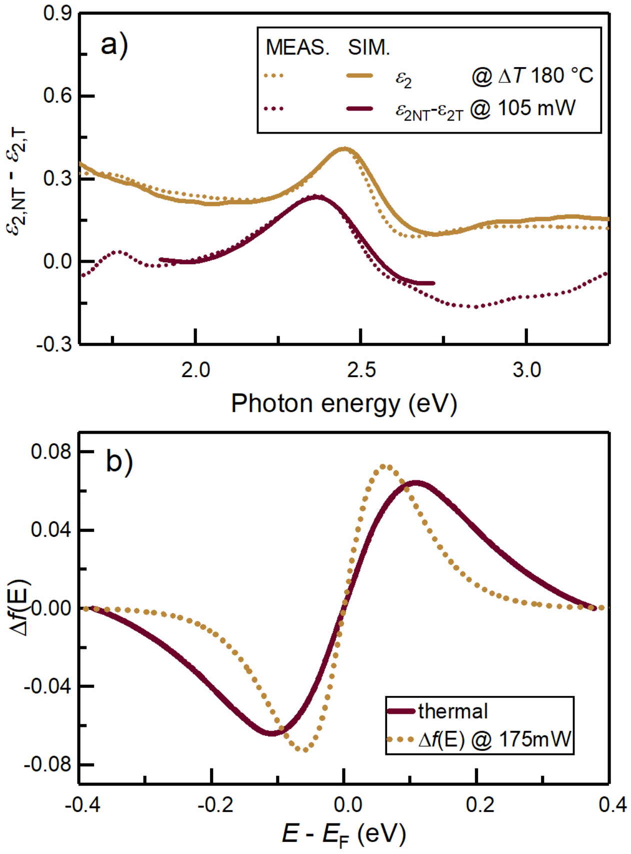

Fig. S9. a) $\Delta\varepsilon_2$ curves for our measurements involving SPP excitation and for a pure thermal case. b) The corresponding changes in the electron distributions.

References:

[1] B. Johs, J.S. Hale, “Dielectric function representation by B-splines,” Phys. Status Solidi A **205**, 715-719 (2008).

[2] P. B. Johnson and R. W. Christy, “Optical constants of the noble metals,” Phys. Rev. B **6**, 4370-4379 (1972).

[3] K. M. McPeak, S. V. Jayanti, S. J. P. Kress, S. Meyer, S. Iotti, A. Rossinelli, and D. J. Norris, “Plasmonic films can easily be better: Rules and recipes,” ACS Photonics **2**, 326-333 (2015).

[4] R. L. Olmon, B. Slovick, T. W. Johnson, D. Shelton, S.-H. Oh, G. D. Boreman, and M. B. Raschke, “Optical dielectric function of gold,” Phys. Rev. B **86**, 235147 (2012).

[5] M. Magnozzi, M. Ferrera, L. Mattera, M. Canepa, F. Bisio, “Plasmonics of Au nanoparticles in a hot thermodynamic bath,” Nanoscale **3**, 1140 (2019).

[6] database of Woollam WVASE software
[7] H. Reddy, U. Guler, A. V. Kildishev, A. Boltasseva, and V. M. Shalaev, "Temperature dependent optical properties of gold thin films," Opt. Mat. Exp. **6**, 2776 (2016).
[8] H. Fujiwara, Spectroscopic Ellipsometry: Principles and Applications (John Wiley & Sons, Ltd, 2007).
[9] T. Favaloro, J.-H. Bahk and A. Shakouri, "Characterization of the temperature dependence of the thermoreflectance coefficient for conductive thin films," Rev. Sci. Ins. **86**, 024903 (2015).
[10] R. B. Wilson, B. A. Apgar, L. W. Martin, and D. G. Cahill, "Thermoreflectance of metal transducers for optical pump-probe studies of thermal properties," Opt. Express **20**, 28829 (2012).
[11] M. G. Burzo, P. L. Komarov, and P. E. Raad, "Optimized Thermo-Reflectance System for Measuring the Thermal Properties of Thin-Films and Their Interfaces," 22nd IEEE SEMI-THERM Symposium, DOI: 10.1109/STHERM.2006.1625211
[12] A. Beran, "The Reflectance Behaviour of Gold at Temperatures up to 500°C," TMPM 34, 211-215 (1985).
[13] R. W. Boyd, Z. Shi, and I. De Leon, "The third-order nonlinear optical susceptibility of gold," Opt. Comm. **326**, 74 (2014).
[14] T. V. Teperik, P. Nordlander, J. Aizipurua, and A. G. Borisov, "Quantum effects and nonlocality in strongly coupled plasmonic nanowire dimers," Opt. Express, 21, 27306, (2013).
[15] D. Forcella, C. Prada, and R. Carminati, "Causality, Nonlocality, and Negative Refraction," Phys. Rev. Lett. **118**, 134301 (2017).
[16] N. E. Christensen and B. O. Seraphin, „Relativistic band calculation and the optical properties of gold," Phys. Rev. B **4**(10), 3321 (1971).
[17] T. Heilpern, M. Manjare, A. O. Govorov, G. P. Wiederrecht, S. K. Gray, and H. Harutyunyan, "Determination of hot carrier energy distributions from inversion of ultrafast pump-probe reflectivity measurements," Nat. Comm. 9, 1853 (2018).
[18] M. Guerrisi, R. Rosei, P. Winsemius, "Splitting of the interband absorption edge in Au," Phys. Rev. B 12(2), 557 (1975).
